# Increased RNA virus population diversity improves adaptability

**DOI:** 10.1038/s41598-021-86375-z

**Published:** 2021-03-25

**Authors:** Florian Mattenberger, Marina Vila-Nistal, Ron Geller

**Affiliations:** 1 Institute for Integrative Systems Biology, I2SysBio (Universitat de València-CSIC), C. Catedràtic José Beltrán 2, 46980 Paterna, Spain; 2 Present Address: Department of Physiology, Genetics and Microbiology, Universidad de Alicante, C. San Vicente del Raspeig s/n, 03690 Alicante, Spain

**Keywords:** Virology, Experimental evolution

## Abstract

The replication machinery of most RNA viruses lacks proofreading mechanisms. As a result, RNA virus populations harbor a large amount of genetic diversity that confers them the ability to rapidly adapt to changes in their environment. In this work, we investigate whether further increasing the initial population diversity of a model RNA virus can improve adaptation to a single selection pressure, thermal inactivation. For this, we experimentally increased the diversity of coxsackievirus B3 (CVB3) populations across the capsid region. We then compared the ability of these high diversity CVB3 populations to achieve resistance to thermal inactivation relative to standard CVB3 populations in an experimental evolution setting. We find that viral populations with high diversity are better able to achieve resistance to thermal inactivation at both the temperature employed during experimental evolution as well as at a more extreme temperature. Moreover, we identify mutations in the CVB3 capsid that confer resistance to thermal inactivation, finding significant mutational epistasis. Our results indicate that even naturally diverse RNA virus populations can benefit from experimental augmentation of population diversity for optimal adaptation and support the use of such viral populations in directed evolution efforts that aim to select viruses with desired characteristics.

## Introduction

RNA viruses are characterized by extreme mutation rates that are orders of magnitudes higher than those of most DNA-based organisms^1,2^. Together with their short replication times and large population sizes, these high mutation rates confer RNA viruses an extreme capacity for rapid evolution. This, in turn, poses a significant challenge for treating and preventing infections by RNA viruses as it allows for subverting the immune system, gaining resistance to antiviral drugs, and jumping to new hosts. On the other hand, the capacity of RNA viruses to rapidly adapt to new environments can be capitalized upon to select viruses with desired characteristics. In such directed evolution experiments, virus populations are grown under conditions that favor either the emergence or further optimization of the desired phenotype. For example, this process has been used to obtain live attenuated vaccines for numerous viruses by repeated growth at suboptimal conditions^3^, improving in vivo models by serial infection of the desired host^4–9^, isolating RNA viruses with high-fidelity polymerases by growth under condition of increased mutational load^10,11^, selection of viruses with improved oncolytic properties^12–14^, or isolation of viruses and virus-like particles of increased stability^15–17^. To date, these studies have relied upon the natural mutational processes of RNA viruses to generate the diversity upon which selection is applied.

As a consequence of degeneracies in the genetic code, single mutations within a codon can on average reach 5.8 (CI_95_ 5.61–6.00) other amino acids while double and triple mutations are required to reach the remaining 9.61 (CI_95_ 9.28–9.93) and 3.59 (CI_95_ 3.24–3.94) amino acids, respectively (Table S1). While the mutation rates of RNA viruses are high, the probability that multiple mutations occur in the same codon remains low. For example, for a virus with a protein-coding region of 6.5 kb (e.g. the picornavirus coxsackievirus B3) and a mutation rate of 1 × 10^–4^^18,19^, the probability that two or three mutations occur in the same codon is 6.8 × 10^–5^ and 6.8 × 10^–9^, respectively (Binomial distribution; see https://github.com/RGellerLab/dms_thermal_selection for calculation). Hence, extremely large population sizes are required to sample the full spectrum of amino acid mutations across the viral protein-coding region. Moreover, inherent preferences in base misincorporation by the polymerase, such as the high transition to transversion bias frequently observed in RNA viruses^20,21^, can further reduce the probability of certain mutations occurring. While the sequential acquisition of mutations over several replication cycles can potentially allow for multiple mutations within a codon to accumulate, selection against intermediate genotypes can limit such evolutionary trajectories. The viral genotype can also influence the probability of sampling particular non-synonymous mutations during replication as synonymous codon choice can influence the likelihood of reaching particular amino acid changes^22^ and dictate evolutionary trajectories^23,24^. In sum, despite the high mutation rate of RNA viruses, the ability of RNA virus populations to reach particular non-synonymous mutations that may be beneficial for adaptation can require extremely large population sizes and can depend on the initial viral genotype. This raises the question of whether directed evolution experiments can benefit from the use of viral populations with experimentally increased population diversity rather than solely relying on the spontaneous emergence of mutations during virus replication.

In this work, we apply a codon-level mutagenesis protocol to the entire capsid region of the human picornavirus coxsackievirus B3 (CVB3) and generate viral populations with increased diversity across the capsid protein. Using these CVB3 populations, we examine if RNA viruses can further benefit from an initial increase in diversity to adapt to a single selection pressure, thermal inactivation of the capsid. We find that in an experimental evolution setting, high diversity CVB3 populations achieve greater adaptation, with a significantly improved ability to resist thermal inactivation compared to standard populations. Additionally, we identify several mutations in the CVB3 capsid that allow for resistance to thermal inactivation and find that mutational epistasis plays an important role in adaptation. Overall, our results indicate that even RNA viruses with extreme mutation rates can further benefit from augmentation of population diversity for adaptation, and support the use of such viral populations in directed evolution experiments.

## Results

### Generation of CVB3 populations with increased diversity across the capsid

**Figure 1:**
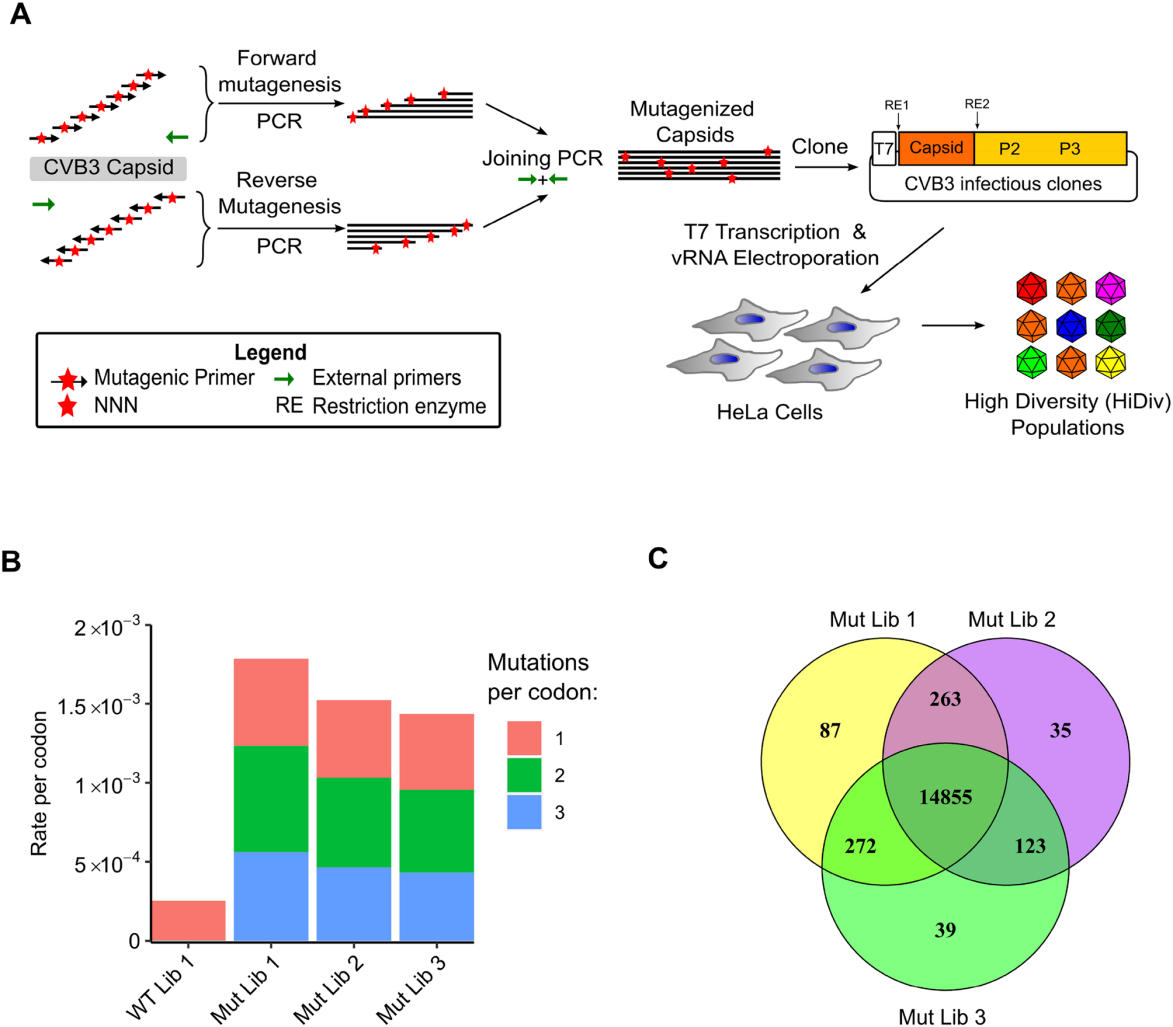
Codon-level mutagenesis of the CVB3 capsid. (**A**) Schematic representation of the mutagenesis protocol. A forward mutagenesis PCR reaction was performed using a single external reverse primer and a pool of forward mutagenic primers targeting each codon in the capsid, each primer encoding degenerate nucleotides (NNN) at the codon matching position. Similarly, a reverse mutagenesis PCR reaction was performed using a mix of reverse mutagenic primers targeting all codon sites in the capsid and a single external forward primer. The product of these PCRs was joined using the external primers and cloned into the CVB3 infectious clone to generate the mutagenized libraries. Viral genomic RNA (vRNA) was produced via in vitro transcription and electroporated into cells to generate high diversity CVB3 populations (HiDiv). (**B**) The mutation rates for single, double, and triple mutations within codons observed in the control, unmutagenized library (WT Lib 1) or the mutagenized libraries (Mut Lib 1–3). (**C**) Venn diagram showing the number of amino acid mutations observed in the mutagenized libraries.

Our aim was to examine how initial population diversity influences adaptation to a single selection pressure, comparing standard CVB3 populations with those harboring experimentally increased diversity. We chose to increase diversity across the 851 amino acid capsid region (P1) of CVB3 as it is not involved in viral genome replication and therefore will not introduce any bias in the capacity of the virus to evolve. For this, a PCR-based method for introducing mutations at the codon level^25,26^ was performed in triplicate to produce three independent libraries of the CVB3 infectious clone harboring increased diversity in the capsid region (see “Methods”; Fig. 1A). Sanger sequencing of 40 clones from the three libraries (34,040 codons; range 7659–15,318 per library) indicated an average mutation rate of 1.1 codon mutations per clone (range 1.06–1.23; Table S2) while sequencing of four clones from a control, non-mutagenized library revealed no mutations (p < 0.05 by Fisher’s exact test). The majority of clones showed either no mutation (30%) or a single codon mutation (45%), while only 25% of clones had > 1 mutation (Fig. S1A), and the number of mutations per codon showed a nearly even distribution (Fig. S1B). We next employed a high-fidelity next-generation sequencing technique to better define library diversity^27^. A high rate of background mutations was observed for single mutations within codons compared to a non-mutagenized library (WT Lib; Fig. 1B; Table S3), while two or three mutations per codon showed > 400-fold higher rates in the mutagenized libraries compared to the non-mutagenized library (Fig. 1B; Table S3). Hence, we chose to exclude single mutations per codon from our dataset as these could not be readily distinguished from background errors. Analyzing only double and triple mutations per codon, we observe an average of 0.9 codon mutations per capsid region and a total of 92% of all possible single amino acid mutations represented in all three libraries (14,855 of 16,169 possible mutations; Fig. 1C). Hence, our mutagenized libraries capture the vast majority of possible single amino acid mutations in the capsid region.

### Selection for viral populations with increased resistance to thermal inactivation

The mutagenized libraries were then used to produce high diversity (HiDiv) viral populations by electroporation of in vitro transcribed viral genomic RNA into HeLa-H1 cells (vRNA; Fig. 1A). Infection was stopped following nine hours to allow for only a single infection cycle. As the initial multiplicity of infection was unknown, a second round of infection was performed to link each capsid to the genome it encapsidates. WT populations were produced in the same manner and grown for two additional passages to expand viral diversity. We recently published an in-depth analysis of the mutations present in similarly produced mutagenized (e.g. HiDiv populations) and WT viral populations^26^, validating the ability of this experimental approach to generate viral populations with increased diversity compared to standard WT virus populations.

**Figure 2:**
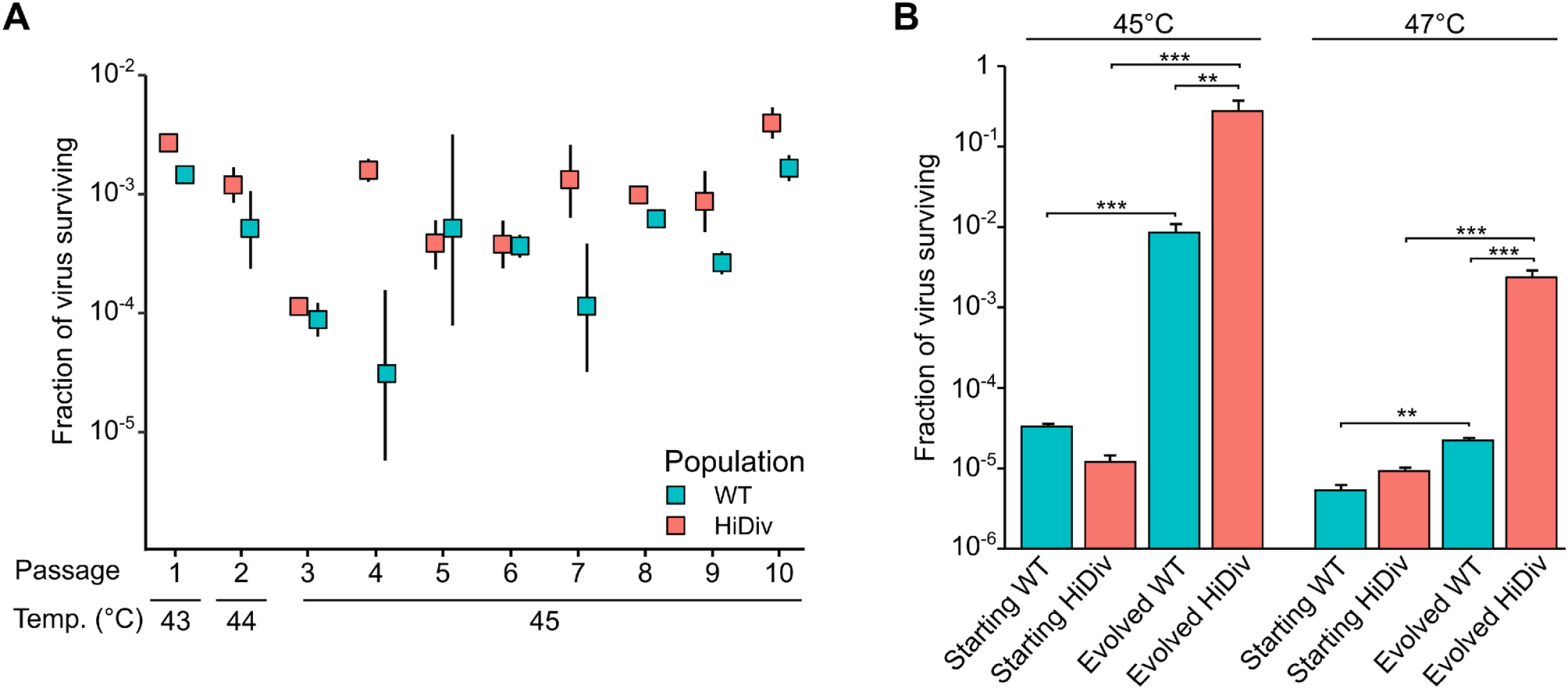
Experimental evolution regime for selection of resistance to thermal inactivation. (**A**) Experimental evolution of HiDiv and WT populations under conditions of thermal inactivation. HiDiv or WT populations were subjected to heat treatment at the indicated temperature for 30 min. The surviving fraction was then amplified and used for the subsequent passage. This process, representing a single passage, was repeated a total of ten times at the indicated temperature. The mean fraction of virus surviving and standard deviation for the three replicates are plotted. (**B**) HiDiv populations show improved resistance to thermal inactivation compared to WT populations. The starting (passage 0) and the evolved (passage 10) HiDiv and WT populations were tested for their ability to resist thermal inactivation following incubation at 45 °C or 47 °C for 30 min. Each population was tested in triplicate and the average and standard deviation of the fraction of surviving viruses is indicated. *p < 0.05, **p < 0.01 ***p < 0.001 by two-tailed t-test on log-transformed values.

To compare the effect of increased initial population diversity on adaptation, the three HiDiv and three WT viral populations were then subjected to an experimental evolution regime to select for thermal resistance. For each passage, approximately one million plaque-forming units (PFU) were heated for 30 min and the surviving viruses were used to inoculate HeLa-H1 cells. Infections were stopped once significant cytopathic effect was observed in most conditions in order to minimize differences in the number of replication cycles between the different conditions. This protocol was repeated for a total of ten passages (Fig. 2A). An initial inactivation temperature of 43 °C was chosen to minimize bottlenecking, followed by a passage at 44 °C, and then eight passages at 45 °C. At each step, the titers of the initial inoculum, the surviving population, and the amplified population were determined and used to calculate the fraction of surviving viruses (Fig. 2A and Table S4). Overall, no major bottlenecks were observed for any of the passages except for passage 4 of the WT line 3 virus population, where only 5 PFU survived the inactivation (Table S4).

To examine whether the evolved populations achieved increased thermal resistance compared to the starting populations, we tested the ability of both the starting (passage 0) and evolved (passage 10) populations to survive an equivalent thermal stress to that applied during the experimental evolution regime (45 °C) or a stronger selection pressure (47 °C). As expected, both the HiDiv and WT populations gained significant resistance to thermal inactivation at both temperatures following the ten passages (Fig. 2B). On average, the fraction of viruses surviving incubation at 45 °C increased by > 20,000 times for the HiDiv population and 256 times for the WT population relative to their respective starting populations (p < 0.001 for both HiDiv and WT by a two-tail t-test; Fig. 2B and Table S5). Similarly, an increase of 255 and 2.3 times in the viruses surviving treatment at 47 °C was observed for the evolved HiDiv and WT populations versus their starting populations, respectively (p < 0.001 and p < 0.01 by a two-tail t-test; Fig. 2B and Table S5). Importantly, the evolved HiDiv populations showed significantly increased thermal resistance compared to the evolved WT population, yielding 33 and 127 times more viruses on average at 45 °C and 47 °C, respectively (p < 0.01 and p < 0.001 by a two-tail t-test; Fig. 2B and Table S5). Hence, the increased initial diversity of the HiDiv populations improved adaptation to the applied selection pressure and conferred a higher capacity to confront stronger selection pressures.

### Identification of novel mutations conferring thermal resistance

To identify mutations that confer resistance to thermal inactivation, the capsid sequence of all adapted populations were obtained by Sanger sequencing at both passages 5 and 10. At passage 10, numerous mutations were observed in the populations subjected to thermal selection, none of which were observed in a mock selected WT population (Table 1 and Table S6). Overall, HiDiv populations had a larger number of mutations compared to WT populations (p < 0.05 by the Mann–Whitney test; Table 1 and Table S6). Mutations were found largely at variable positions in CVB capsids (p < 0.005 by Mann–Whitney test; Fig. S2 and Table S7). In total, nine mutations were observed in at least two independent lines, indicating these may contribute to thermal stability. Most of these mutations were in loops and subunit interfaces, as expected for capsid stabilizing mutations (Tables S6 and S7). The A512T mutation (see Table 1 for the position of mutations within the individual capsid proteins), which was previously reported to confer capsid stability following prolonged incubation at 37 °C^28^, was observed in all 3 WT lines and 2 of 3 HiDiv lines. In addition, different mutations at position 581 were present in all evolved lines, indicating this position may be particularly relevant for thermal stability.

**Table 1:** The high-frequency mutations observed at passage 5 and 10 of the experimental evolution regime.

Capsid protein	Wildtype AA	CDS site	Mutant AA	Protein position	Passage 5							Passage 10						
					Mock	WT			HiDiv			Mock	WT			HiDiv		
					1^a^	1	2	3	1	2	3	1^a^	1	2	3	1	2	3
VP4	G	51	S	51														×
VP2	G	162	R/W	93													×	
	H	187	R	118													×	
	L	206	P	138					×		×							
	D	207	S	138					×							×	×	
VP3	N	395	H	63		×	×						×	×				
	A	512	T	180		×	×						×	×	×		×	×
	Q	566	L	234	×							×						
VP1^b^	**A**	**576**	**T**	**6**											**×**			
	**A**	**576**	**V**	**6**					**×**							**×**	**×**	
	**I**	**581**	**K**	**11**		**×**	**×**						**×**					
	**I**	**581**	**M**	**11**				**×**		**×**					**×**			
	**I**	**581**	**T**	**11**					**×**	**×**	**×**			**×**		**×**	**×**	**×**
	E	596	G	26					×							×	×	
	I	634	V	64						×								
	F	646	Y	76		×	×						×	×				
	I	711	V	141				×		×					×		×	
	V	720	I	150						×								
	Q	824	G	254				×		×					×			×
	K	827	R	257								×						
	K	829	Q	259					×							×		

^a^WT viral population passaged in HeLa-H1 cells without heat treatment.

^b^Bold font indicates positions where multiple non-synonymous mutations were observed.

**Figure 3:**
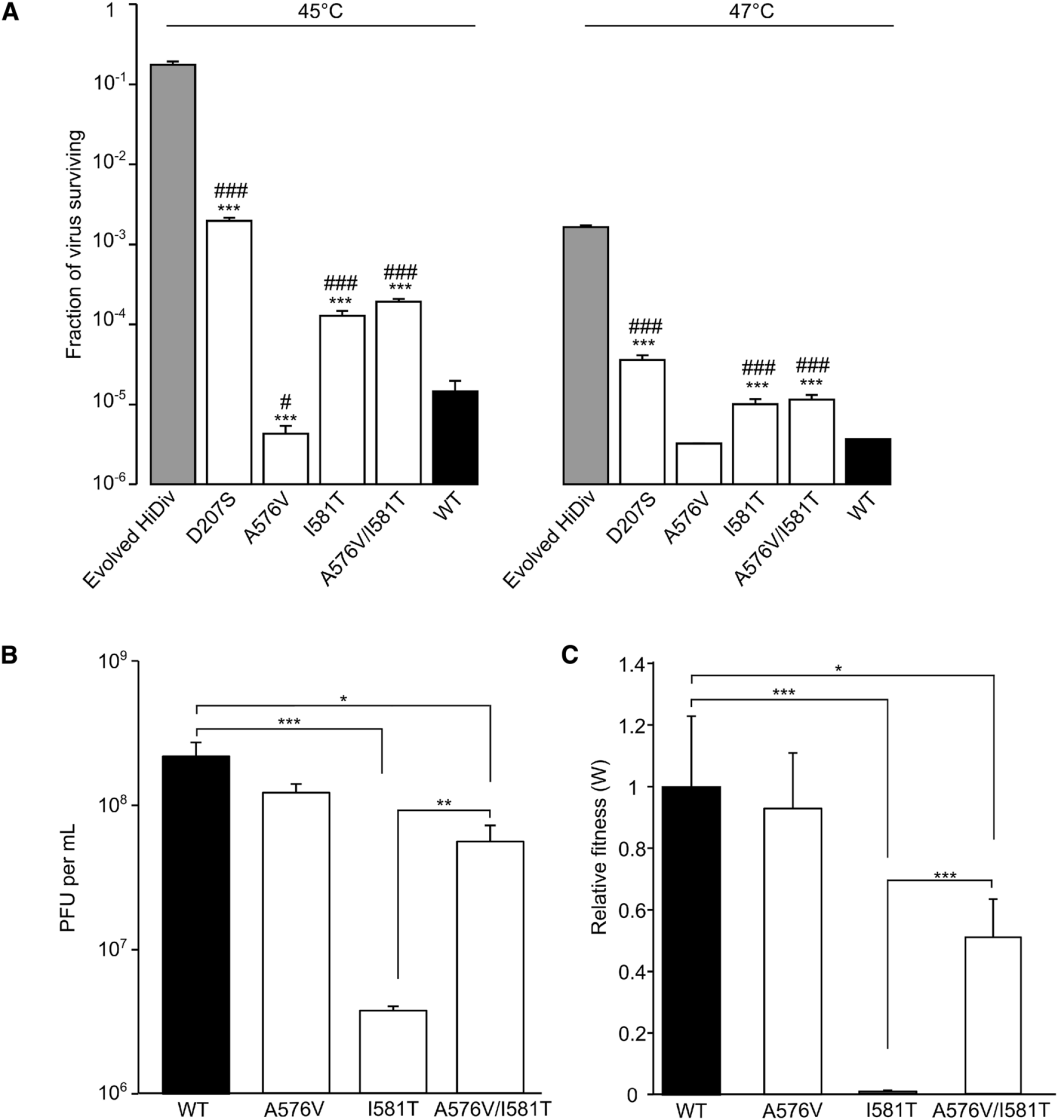
Resistance to thermal inactivation of select mutants observed in the evolved HiDiv populations. (**A**) The fraction of virus surviving thermal treatment at the indicated temperature for the different viral populations. A value of 1 (the limit of detection) was added to conditions where no viruses survived thermal inactivation to enable graphical and statistical analysis. (**B**) Virus yield following a single cycle of replication for the different viral populations (PFU, plaque-forming units). (**C**) The relative fitness of the different viral populations obtained using a direct competition assay. The mean and SEM of at least three independent replicates is plotted. *^/#^p < 0.05, **^/##^p < 0.01, ***^/###^p < 0.001 by a two-tailed t-test on log-transformed data versus the starting WT (*) or evolved HiDiv (#) populations.

Due to the higher thermal resistance of capsids from the HiDiv populations, we chose to experimentally test all mutants observed in more than one HiDiv line for their ability to confer thermal stability. However, we did not evaluate the effect of A512T, as this has already been demonstrated to enhance capsid stability following prolonged incubation at 37 °C^28^. All tested mutations except for the A576V mutation showed higher thermal resistance than the starting WT populations at both 45 °C and 47 °C (p < 0.001 by two-tailed t-test versus the starting WT population; Fig. 3A and Table S8). Surprisingly, the A576V mutation reduced thermal resistance by 3.4 fold compared with the starting WT population at 45 °C (p < 0.05 by two-tailed t-test; Fig. 3A and Table S8) and yielded no viable viruses at 47 °C. Hence, this mutation was not involved in thermal resistance despite being present in two of the three evolved HiDiv populations and one of the three evolved WT populations at passage 10 (Table 1). On the other hand, no single mutation was sufficient to mirror the phenotype of the evolved HiDiv population at 45 °C or 47 °C, indicating optimal thermal resistance requires multiple synergistic mutations (Fig. 3A and Table S8). From the individual mutations analyzed, D207S showed the strongest effect on thermal inactivation, yielding 163 and 12 times more surviving viruses compared to the WT virus following incubation at 45 °C and 47 °C, respectively (p < 1 × 10^–6^ by two-tailed t-test for both temperatures; Table S8). The I581T mutation (present in all HiDiv and 1 WT evolved populations) showed a more modest increase in thermal resistance compared to the WT virus, conferring an increase in the number of viruses surviving heat treatment of 18 or 6 times for 45 °C and 47 °C, respectively (p < 1 × 10^–4^ and p < 1 × 10^–5^ by two-tail t-test; Table S8). Interestingly, we could not produce viral populations harboring the E596G mutation in three independent attempts, indicating a strong fitness cost for this mutations when in isolation and highlighting the role of epistasis in the evolved lines.

Since the A576V mutation was found to confer thermal sensitivity rather than thermal resistance (Fig. 3A and Table S8), we hypothesized it may act synergistically with another mutation to improve either thermal resistance or viral fitness. Indeed, this is supported by the fact that A576V was always observed in the context of the I581T mutations. We first examined whether the double mutant (A576V/I581T) showed an improved ability to resist thermal inactivation compared to the A576V or I581T single mutants (Fig. 3A). While significantly more thermal resistant than A576V alone (p < 0.001 at 45 °C by two-tailed t-test), the double mutant showed a similar ability to resist thermal inactivation compared to the I581T mutant (p > 0.05 at 45 °C and 47 °C by two-tailed t-test), indicating that their co-occurrence was not due to selection for improved thermal resistance (Fig. 3A and Table S8). We next assessed the replicative fitness of the individual mutants or the double mutant. The A576V mutation did not alter virus yield compared to the WT virus following a single replication cycle, indicating a neutral effect on fitness under normal growth conditions (p > 0.05 using a two-tail t-test; Fig. 3B and Table S9). In contrast, the I581T mutation had a strong cost to fitness, producing 58 times less infectious particles compared to the WT virus (p < 0.001 using a two-tail t-test; Fig. 3B and Table S9). The A576V/I581T double mutant showed only a modest, three-fold reduction in virus yield compared to the WT virus, indicating that A576V compensates for the fitness cost incurred by the thermal resistant I581T mutation (p < 0.05 using a two-tail t-test; Fig. 3B and Table S9). To validate this finding, we directly assessed the relative fitness of the single and double mutants compared to the WT virus using a highly-sensitive qPCR-based competition assay (Fig. 3C and Table S10). As before, the A576V mutation did not reduce viral fitness appreciably (p > 0.05 by a two-tail t-test; Fig. 3C), while the I581T mutation resulted in a strong fitness cost (> 150-fold reduction, p < 0.01 by two-tailed t-test; Fig. 3C), which was largely compensated for in the double A576V/I581T mutant (~ twofold reduction, p > 0.05 by two-tailed t-test; Fig. 3C).

### The evolutionary pathway to thermal resistance

To better understand the evolutionary pathway to achieve thermal resistance, we compared the mutations present at passage 5 to those observed at passage 10 (Table 1). All evolved populations had mutations at position 581 by passage 5, which were maintained at passage 10, indicating a central role for this position in thermal resistance. Of the three different mutations observed at this position (I581T/K/M), I581T was observed in three of the six evolved populations at passage 5 and four of six populations at passage 10, suggesting it confers a higher fitness gain. This is further supported by the conversion of an I581K mutation at passage 5 to an I581T mutation by passage 10. Mutations at position 576, which are likely to compensate for mutations at position 581, as observed in the case of the A576V and I581T mutations examined above (Fig. 3B,C), increased in frequency from a single population at passage 5 to three populations in passage 10. Hence, selection for thermal resistance occurred before the selection of compensatory mutations that restore replicative fitness. The strongly thermal resistant D207S mutation (Fig. 3A) increased in frequency from one population at passage 5 to two at passage 10, while the capsid stabilizing A512T mutation^28^ increased in frequency more drastically, from two to five populations between passages 5 and 10. Other mutations maintained their frequency at both passages (F646Y, I711V, and Q824G), suggesting a neutral or beneficial effect. Finally, mutation E596G, which failed to produce viable virus when introduced in isolation, increased in frequency from one evolved population to two evolved populations during the five additional passages. This mutation was always observed in the context of the D207S, A576V, and I581T mutations, suggesting it may act synergistically with one or more of these mutations. In sum, all evolved lines at passage 10 had at least two mutations that increased thermal resistance independently (I581 with D207S and/or A512T), with compensatory mutations arising after the selection of thermal resistant mutations. Hence, optimal adaptation of resistance to thermal inactivation is complex, requiring multiple changes that either increase thermal resistance or help compensate for reductions in fitness.

## Discussion

RNA viruses have an extreme capacity for rapid evolution due to low-fidelity replication machinery, large population sizes, and small genomes. Indeed, RNA viruses replicate at the brink of error catastrophe, where even small increases in mutation rates can lead to the extinction of the population as a result of too many mutations^29^. Previous studies using high-fidelity polymerase mutants have shown that RNA virus populations with reduced diversity are less able to adapt to new environments compared to standard virus populations^10,11,30^. In the current work, we examine the opposite scenario, testing whether RNA viruses can further benefit from experimental augmentation of initial population diversity during experimental evolution. We chose to increase diversity using a PCR-based, codon-level mutagenesis protocol which, unlike error-prone mutagenesis, allows for the introduction of all possible single amino acid mutations away from the original viral genotype. We find that experimentally increasing initial population diversity enables viral populations to achieve better adaptation to the selection regime (HiDiv versus WT population at 45 °C; Fig. 2B). Interestingly, not only were HiDiv populations better adapted to survive inactivation at the temperature confronted during the experimental evolution regime, they were also better able to survive a stronger selection pressure (HiDiv versus WT population at 47 °C; Fig. 2B). Overall, these results indicate that even RNA viruses with high mutation rates can benefit from augmentation of population diversity to increase their capacity to adapt. While we investigated adaptation to a single selection pressure, increasing initial population diversity may be of particular relevance for adaptation to more complex environments such as changes in host species. Overall, these findings have implications for directed evolution experiments aimed at the selection of desired traits in RNA viruses, such as increased stability of live attenuated vaccines, alterations of tissue tropism in gene therapy, or optimization of oncolytic activity.

Analysis of mutations present in the adapted populations following 5 and 10 rounds of selection revealed numerous non-synonymous changes in both the WT and HiDiv populations (Table 1 and Table S6), indicating that optimal adaptation to thermal stability requires multiple mutations in the capsid. Mutations at position 581 were present in all populations by passage 5, suggesting these are key for adaptation to thermal stability. On the other hand, the A512T mutation, which was previously shown to confer stability during prolonged incubation at 37 °C^28^, and therefore is likely to also be stabilizing at higher temperatures, only reached high frequency at passage 10. In addition, we observed mutations that conferred thermal sensitivity (A576V) or which failed to yield infectious virus when present individually (E596G), indicating strong epistatic interactions that are common in RNA viruses^31^ (Fig. 3). This is exemplified by mutations at positions 576 and 581. Mutation A576V was more sensitive to thermal inactivation relative to the WT virus but had no significant cost to fitness (Fig. 3B,C). The I581T mutation, on the other hand, was more resistant to thermal inactivation but incurred a significant fitness cost (Fig. 3B,C). In combination, the A576V/I581T double mutant showed similar resistance to thermal inactivation as the I581T mutation alone but with a significantly lower cost to fitness (Fig. 3B,C). Interestingly, the I581M mutation, which did not yield viable virus when in isolation (data not shown), occurs in the context of a different mutation at position 576 (A576T), suggesting an even stronger epistatic interaction between these two mutations.

From the mutations shown to confer resistance to thermal inactivation, the D207S mutation found in two of the three HiDiv populations conferred the greatest resistance to thermal inactivation (Fig. 3A). This mutation requires three transversions to occur within the same codon (GAC to TCG; Table S6). The chance for such a mutation to occur during natural viral replication is very low due to both the reduced probability of multiple mutations occurring in the same codon and the lower rate of transversions compared to transitions in RNA viruses^20,21^. This mutation is also unlikely to have appeared if random mutagenesis methods (e.g. error-prone PCR) were used to enrich the initial population diversity instead of the codon-based mutagenesis strategy employed in this work because the probabilistic nature of mutations inserted by such methods is unlikely to yield multiple mutations within the same codon. Interestingly, the D207S mutation can also be reached by two transition mutations (GAC to AGC), yet this mutation was not observed in the WT evolved populations despite its simpler evolutionary pathway. Other mutations that increase thermal stability were reached by single mutations. This includes the I581T mutation, which was present in all HiDiv populations and conferred a more modest resistance to thermal inactivation (Fig. 3A), as well as the A512T mutation, which was present in five of the six lines. Hence, these mutations are likely to represent more frequent paths for adaptation in natural CVB3 populations, yet may not provide the optimal degree of resistance to thermal inactivation that can be reached by the incorporation of multiple mutations within a single codon.

The high rate of recombination in positive-strand RNA viruses such as CVB3 enables the shuffling of mutations from different genomes, promoting the joining of beneficial mutations in the same genotype while helping to purge deleterious mutations^32^. The large number of mutations observed in all lines after ten passages is likely to arise at least in part from recombination between different genomes. In this sense, recombination is likely to play a key role in the adaptation of these highly diverse populations, especially considering the epistatic nature of the mutations observed in the evolved populations. The availability of mutants in RNA polymerases that show reduced recombination rates^33,34^ can provide an interesting tool for dissecting the role of recombination in the ability of highly diverse populations to better adapt to new environments compared to standard populations.

## Methods

### Viruses, cells, and plaque assays

HeLa-H1 (CRL-1958) and HEK293 (CRL-1573) cells were obtained from ATCC. Cells were cultured in culture media (DMEM with 10% heat-inactivated FBS, Pen-Strep, and L-Glutamine). For infections, FBS concentrations were reduced to 2%. For plaque assays, serial dilutions of the virus were used to infect confluent HeLa-H1 cells in 6 well plates for 45 min, followed by overlaying the cells with a 1:1 mixture of 56 °C 1.6% agar (Arcos Organics 443570010) and 37 °C 2× DMEM with 4% FBS. Two days later, a 10% formaldehyde solution was added to reach a final concentration of 2% to fix the cells and inactivate the virus, followed by staining with crystal violet and counting of plaques. The CVB3 Nancy infectious clone and a marked reference CVB3 infectious clone encoding four adjacent silent mutations in the polymerase for use in the qPCR-based competition assays^35^ (see below) were a kind gift of Marco Vignuzzi (Institut Pasteur).

### Codon-level mutagenesis protocol

Mutagenesis was performed using degenerate primers as previously described^26^ with the exception that two rounds of mutagenesis were performed using 10 cycles and then seven cycles for all samples. The products were gel purified and ligated to an XhoI and Kpn2I digested and gel purified pCVB3-Xho-P1-Kpn2I^26^ using NEBuilder HiFi DNA Assembly reaction (NEB) for 60 min. Mutagenesis efficiency was evaluated by the transformation of the assembled plasmids into NZY5α competent cells (NZY Tech), Sanger sequencing of several clones, and analysis using the Sanger Mutant Library Analysis script (https://github.com/jbloomlab/SangerMutantLibraryAnalysis). Subsequently, the assembled plasmid reactions were purified using a Zymo DNA Clean & Concentrator-5 kit (Zymo Research) and used to electroporate MegaX DH10B T1R Electrocomp cells (ThermoFisher) using a Gene Pulser XCell electroporator (BioRad) according to the manufacturer’s protocol. Cells were then grown overnight in a 10 mL liquid culture at 33 °C. Transformation efficiency was estimated by plating a small amount of the transformation on agar plates. In total, 4.75 × 10^5^, 1.4 × 10^5^, and 7.1 × 10^5^ transformants were obtained for lines 1, 2, and 3, respectively. Of these, 75% contained full-length inserts as judged by colony PCR of 52 colonies using external primers 659F (TTGGATTGGCCATCCGGT) and 3450R (GTGCTGTGGTCGTGCTCACTAA). Subsequently, plasmid DNA was isolated in duplicate using a miniprep kit (Macherey–Nagel NucleoSpin Plasmid).

### Analysis of mutagenized libraries

To analyze library diversity, the mutagenized region was amplified from 10 ng of each library or the unmutagenized control plasmid by performing 25 PCR cycles using Phusion polymerase and the HiFi-F (CTTTGTTGGGTTTATACCACTTAGCTCGAGAGAGG) and HiFi-R (CCTGTAGTTCCCCACATACACTGCTCCG) primers, followed by gel purification of the correct band (Zymoclean Gel DNA Recovery Kit). Libraries were then prepared following published protocols^36^ and each library was run on a Novaseq6000 2 × 150 at a maximum of 30G per lane to reduce potential index hopping. Analysis of mutations was performed as previously published^26^.

### Production of WT and high diversity viral populations

To produce viral populations, viral genomic RNA was transcribed from SalI linearized plasmids (TranscriptAid T7, ThermoScientific) and 10 μg were electroporated into 4 × 10^6^ cells in a 4 mm cuvette in 400 μL of calcium and magnesium-free PBS using a Gene Pulser XCell (BioRad) set to 240 V and 950 μF. Two electroporations were performed for each mutagenized library. Cells were then placed in culture media for 9 h to produce the passage 0 virus (P0). Following two freeze–thaw cycles, 2 × 10^6^ plaque-forming units (PFU) were used to infect a 90% confluent 15 cm plate in 3 mL of infection media for 45 min. Cells were then washed with PBS and incubated in 13 mL of infection media for 8 h. Finally, cells were subjected to 3 freeze–thaw cycles, debris removed by centrifugation at 500xg and the supernatants collected to generate P1 virus stocks. All infections produced > 3.75 × 10^7^ PFU in P0 and > 2 × 10^8^ PFU in P1 as judged by plaque assay. Wildtype virus was similarly produced by electroporation of viral RNA into cells. Viral infections were allowed to continue until CPE was observed to generate the P0 population. The cultures were then subjected to two freeze–thaw cycles, followed by titration. Two additional passages at low MOI were performed to enable the natural accumulation of diversity generated by the viral polymerase and avoid any biases that may be introduced by the T7 RNA polymerase used in the in vitro transcription of viral RNA.

### Experimental evolution for thermal resistance

Viral populations were adjusted to ~ 10^7^ PFU/mL and two aliquots of 100 μL were subjected to the indicated temperature for 30 min in a 0.2 mL PCR tube using a thermocycler. Both an unheated virus population and one of the heated aliquots were titered using a plaque assay to assess the degree of infectivity loss. The remaining aliquot was used to infect cells in a six well plate until cytopathic effect (CPE) was reached in most wells to ensure a similar number of replication cycles between different conditions. The emerging virus was titered and used for the next round of inactivation. To control for mutations conferring adaptation to cells, a WT virus population was blindly passaged for ten passages in a single well of a six well plate. For this, viral titers of 10^8^ PFU/mL were assumed upon CPE and an estimated 1000 PFU from each infection was used to initiate the subsequent passage in order to mimic the amount of virus surviving heat treatment. Sanger sequencing was performed for all populations following ten passages by extracting RNA from 100 μL of virus supernatant (Zymo Quick RNA Viral kit), generating cDNA using a gene-specific primer (TCTCTTGGACCTCTACTA) with M-MLV reverse transcriptase (NZY Tech), amplifying the P1 region with Phusion polymerase and primers 659F (TTGGATTGGCCATCCGGT) and 3548R (TTGGGTAGTGCTTGTTTTTGG), and sequencing the purified PCR product using primers HiFi-F, 2045F (TCGAGTGTTTTTAGTCGGACG), 2143R (GGCCGAACCACAGAACATAA) and 3450R (GTGCTGTGGTCGTGCTCACTAA). Sequences were analyzed using the Staden 2.0.0 package.

### Evaluation of thermal resistance

To evaluate the thermal resistance of the virus populations following ten passages, we performed a heat inactivation experiment at 45 °C and 47 °C. For this, we adjusted the viral populations to 10^6^ PFU/mL and took 100 µL in 0.2 mL PCR tubes. The virus populations were subjected to heat inactivation at the indicated temperature for 30 min in a thermocycler. Both the starting virus inoculum and the surviving virus titer were obtained by plaque assay, and the fraction of the surviving virus was calculated. Experiments were performed in triplicate for each population and the input populations were titered twice to ensure no major bias in the initial amount of virus.

### Generation and evaluation of CVB3 capsid mutants

The PCR of the capsid region used as a template for mutagenesis was phosphorylated and cloned into a SmaI digested pUC19 vector for use in the mutagenesis reactions. For each mutant, non-overlapping primers were designed that incorporated the mutation in the middle of the forward primer. PCR was performed using Phusion polymerase and mutagenic primers, followed by DpnI treatment, phosphorylation, ligation, and transformation of chemically competent bacteria. Successful mutagenesis was verified by Sanger sequencing. Subsequently, the capsid region was subcloned into the infectious clone using XhoI and Kpn2I sites. Plasmids were then linearized with MluI and 1 μg of plasmid was transfected into 2 × 10^6^ HEK293 cells together with 1 μg of a plasmid encoding the T7 polymerase^37^ (Addgene 65974) using Lipofectamine 2000 (ThermoFisher) following the manufacturer's recommendation. Viruses were titered by plaque assay and tested for thermal resistance as indicated above. For mutants that did not yield any virus, the transfection was repeated two more times to ensure the lethality of the mutant. Emerging viral populations were sequenced to ensure no compensatory mutations or reversions arose during replication. For assessing virus production following a single replication cycle, **c**onfluent HeLa-H1 cells in a 6-well plate were infected in triplicate with 3 × 10^6^ PFU for 45 min (multiplicity of infection of 1.5). Subsequently, the inoculum was removed, 2 mL of infection media was added, and the cells were incubated for 9 h. The cells were then subjected to two freeze–thaw cycles and the amount of virus produced was titered using a plaque assay.

### qPCR-based competition assay

The fitness of the mutants was tested by direct competition with a marked reference virus using a previously described TaqMan RT-qPCR method^26,35^. Briefly, in triplicate, confluent HeLa-H1 cells were infected for 45 min with a 1:1 mixture of the mutant and the marked reference virus at a multiplicity of infection of 0.01. Subsequently, the inoculum was removed and the cells were washed twice with PBS. Infection media was then added and the cells were incubated for 24 h at 37 °C. Three freeze–thaw cycles were then performed to release viral particles from cells and the supernatant was centrifuged for 5 min at 300*g*. The supernatant was then treated with DNAseI for 15 min, and RNA was extracted (Quick viral RNA kit, Zymo Research). Quantification of the frequencies of each genome in the completion assay was determined using Luna Universal Probe One-Step RT-qPCR kit (New England BioLabs) containing 3µL of total RNA, 0.4 µM of each primer and 0.2 µM of each probe in a total volume of 10 µL. The standard curve was performed using tenfold dilutions of RNA extracted from 10^7^ PFU of WT and reference virus. Finally, relative fitness (W) of each mutant was determined using the formula W = [R(t)/R(0)]^1/t^, where R(0) and R(t) represent the ratio of mutant and marked reference genomes in the starting inoculum and 24 h after infection (t = 1), respectively**.**

### Bioinformatics and statistical analyses

The crystal structure PDB:4gb3 was used to obtain secondary structure assignment using DSSP and to identify residues present in the core, interface, or surface via the VIPERdb^38^ (http://viperdb.scripps.edu/). Missing residues in the structure were assumed to be flexible loops. Amino acid variability was assessed using Shannon entropy. Briefly, all available, non-identical, full-genome CVB sequences from Virus Pathogen Resource^39^ (www.viprbrc.org) were downloaded (available at https://github.com/RGellerLab/dms_thermal_selection) and codon-aligned using the DECIPHER package in R. All alignment positions not present in our reference strain were removed, and a custom R script was used to calculate Shannon entropy. All statistical tests were performed in R (version 4.0.1) using a two-tail t-test on log-transformed data or a two-sample Mann–Whitney U test via the wilcox.test function, as indicated in the text.

## Supplementary Information

Supplementary Figures.

Supplementary Table S1.

Supplementary Table S2.

Supplementary Table S3.

Supplementary Table S4.

Supplementary Table S5.

Supplementary Table S6.

Supplementary Table S7.

Supplementary Table S8.

Supplementary Table S9.

Supplementary Table S10.

## Data Availability

Unaligned bam files have been uploaded to SRA (Accession SAMN16492169–SAMN16492172, SRX9320721–SRX9320724). The scripts and data required to obtain the codon count tables for all samples, calculating the number of amino acids reached by each type of mutation, calculating the probability of different mutations occurring in the same codon, and the alignment for entropy calculations can be found on Github (https://github.com/RGellerLab/dms_thermal_selection).
