# Supplementary material for: Increased RNA virus population diversity improves adaptability
Source: Sci Rep. 2021 Mar 25;11:6824. doi: 10.1038/s41598-021-86375-z (PMC7994910; doi:10.1038/s41598-021-86375-z)
Supplement: Supplementary file 1 — Supplementary Figures. [file 41598_2021_86375_MOESM1_ESM.pdf]

Increased RNA virus population diversity improves adaptability

Florian Mattenberger, Marina Vila-Nistal, Ron Geller

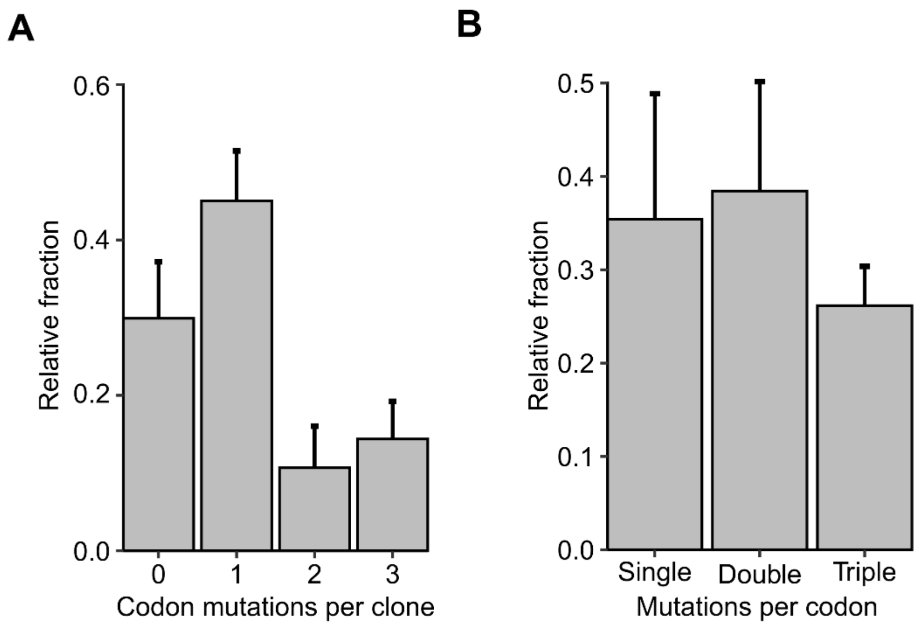

Figure S1. Analysis of library diversity by Sanger sequencing.

A. The average fraction of the number of codon mutations observed per clone in the three mutagenized libraries. **B.** The average fraction of single, double, and triple mutations within each codon mutation in the three mutagenized libraries.

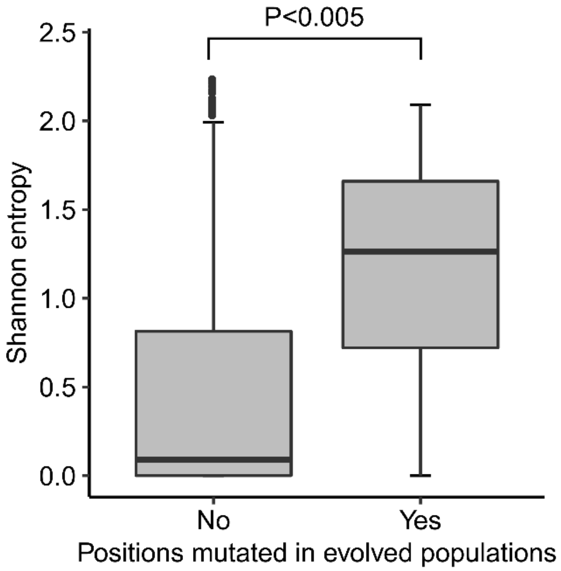

Figure S2. Positions mutated in evolved populations occur in variable positions

Positions where mutations were observed in the thermal selected populations occur at more variable positions in enterovirus B sequences versus all other positions, as judged by Shannon entropy.
